# Relationship Between Post-traumatic Stress Symptoms and Anticipatory Grief in Family Caregivers of Patients With Advanced Lung Cancer: The Mediation Role of Illness Uncertainty

**DOI:** 10.3389/fpsyt.2022.914862

**Published:** 2022-06-09

**Authors:** Di Sun, Zhihui Mao, Xu Zhang, Jiaojiao Li, Lijuan Zhang

**Affiliations:** ^1^School of Nursing, Liaoning University of Traditional Chinese Medicine, Shenyang, China; ^2^School of Nursing, China Medical University, Shenyang, China; ^3^Department of Thoracic Medicine, Cancer Hospital of China Medical University, Liaoning Cancer Hospital and Institute, Shenyang, China

**Keywords:** post-traumatic stress symptoms, anticipatory grief, illness uncertainty, lung cancer survivors, family caregivers

## Abstract

**Objective:**

To explore the interrelationship between post-traumatic stress symptoms (PTSS), illness uncertainty (IU), and anticipatory grief (AG).

**Methods:**

Structural equation modeling with bootstrapping estimation was conducted using data from a convenience sample of 254 family caregivers of patients with advanced lung cancer in China. Participants were recruited from a public cancer hospital in Shenyang, China. The family caregivers completed the Impact of Events Scale-Revised, Uncertainty in Illness Scale Family Caregiver Version, and Anticipatory Grief Scale.

**Results:**

The measurement model has good reliability and validity, and the final model fit the data well. PTSS positively influenced AG (direct effect estimate = 0.391, *p* = 0.002). Moreover, IU was found to mediate the relationship between PTSS and AG (Indirect effects estimate = 0.168, *p* = 0.005). The mediating effect of IU accounted for up to 30.1% of the total effect.

**Conclusion:**

IU mediated the relationship between PTSS and AG. Healthcare professionals should continuously assess PTSS, IU and AG levels in FCs and provide effective intervention options for mitigation.

## Background

Lung cancer is the leading cause of cancer-related morbidity and mortality in China, and according to statistics, the number of newly diagnosed lung cancer cases in 2015 was 787,000 and the number of deaths was 630,500, with lung cancer deaths accounting for 27% of all cancer deaths, equivalent to one out of every four cancer-related deaths (1). The high mortality rate of lung cancer patients in China may be attributed to the lack of routine medical checkups leading to the fact that most Chinese are diagnosed with advanced lung cancer and miss the best time for treatment, thus about two-thirds of patients usually die within 1–2 years (2). Palliative care for patients with advanced lung cancer is often provided by family caregivers (FCs) because the place of death is more likely to be at home than in the hospital due to the traditional Chinese culture and the burden of medical expenses (3). The FCs definition includes any family member, friend, or partner who maintains a significant relationship with the patient and provides some care (4). However, the high mortality rate from advanced lung cancer leads FCs to not only take on heavy care duties but also to manage expectations and emotions associated with the fear of losing someone important to them, a phenomenon known as Anticipatory Grief (AG). AG is defined as the family response to the perceived threat to the other's life and the subsequent anticipation of loss in the context of the end-of-life caregiving relationship (4). A study by Nielsen showed that approximately one-third of FCs experienced AG, with up to 15% of those with severe symptoms (5). While AG is considered a natural progression when caring for a terminally ill relatives, its impacts are nonetheless debilitating for FCs who must learn to cope in the process (6).

Therefore, an increasing amount of attention has been paid to exploring the antecedents of AG, which is required to enhance FCs quality of life and helping them to improve negative emotion. Faced with the imminent loss, FCs react with anxiety, depression, concerns about the future, fear, sadness, feelings of helplessness, compassion fatigue or even Post-traumatic Stress Symptoms (PTSS) (7–11). PTSS is a delayed and persistent psychiatric disorder caused by various traumatic events or catastrophic psychological trauma. The clinical manifestation is a traumatic experience of repeated intrusions into dreams and avoidance of any scenario that might lead to traumatic memories and persistent hypervigilance (12). Previous studies have found that there may be a positive correlation between the level of PTSS experienced by family caregivers and the severity of the patient's illness, and that the level of PTSS experienced by family caregivers is more significant prior to the patient's death (11, 13).

In addition, exposure to PTSS may cause family caregivers to avoid any scenarios that may trigger traumatic memories, such as avoiding information related to the illness, may interfere with their ability to access health-related knowledge and increase their illness uncertainty (IU). IU is defined as “the inability to determine the meaning of illness-related events and accurately anticipate or predict health outcomes” (14). The study we conducted previously confirmed that IU may be an important factor affecting AG (15). IU, also identified as one of the core characteristics in the conceptual framework of AG for FCs, may cause FCs to develop into a permanent state of hypervigilance and traumatic distress toward illness signs, mainly after crisis episodes (4). However, there are few studies that have explored the relationship between the above three variables in depth, resulting in untargeted interventions for AG. Therefore, this study is the first to analyze the development mechanism of AG using a structural equation modeling approach, with the aim of providing a guiding direction for the construction of clinical intervention programs.

The present study was inspired by Stroebe's integrative risk factor framework (16) for the prediction of bereavement outcome. The framework includes various predictors of bereavement outcomes, which work together to describe and determine the sources of individual differences in adjustment to bereavement. In this study, AG as the outcome, PTSS as the loss-oriented stressor, and IU as appraisal process. The study proposed two hypotheses, hypothesis 1 that PTSS is positively related to caregivers AG and hypothesis 2 that IU mediates the effect of PTSS on caregivers AG. The hypothesized model is illustrated in Supplementary Figure 1.

## Method

### Study Design and Participants

This study is a descriptive cross-sectional study. A convenience sample was recruited from a public cancer hospital in Shenyang, China, between January and October 2021. Inclusion criteria were as follows: (1) FCs of patients with a clinicopathological histological or cytological diagnosis of lung cancer and TNM stage III or IV; (2) Age ≥ 18 years; (3) awareness of the patient's disease condition; (4) undertaking the primary care of the patient's daily life and being identified by the patient as their primary caregiver; (5) good reading and communication skills in Chinese; (6) and volunteer for this study. We excluded those who were unable to complete the questionnaire due to psychological or cognitive impairment and those who had <1 month of care. We estimated the sample size according to the requirements of the structural equation modeling (SEM), and the ratio of observed variables to sample size ranged from 1:10 to 1:15, with a sample size between 200 and 400 being appropriate (17). We distributed the survey questionnaires to 298 potential participants and received 254 complete and valid questionnaires out of the 274 possible questionnaires, giving an 92.7% overall response rate. The sample size met the requirements for SEM analysis.

### Data Collection

The study team members were first trained before the start of the study, and a uniform guideline for participants to fill out the questionnaire was clarified to ensure the reliability of the questionnaire. The questionnaire was distributed by members of the study team and completed independently by FCs of patients with advanced lung cancer and consisted of four main sections: Sociodemographic Characteristics of FCs, Impact of Events Scale-Revised, Uncertainty in Illness Scale Family Caregiver Version and the Anticipatory Grief Scale. The assessors collected on the spot the completed questionnaires, and asked the participants to complete any missing options.

### Measures

#### Sociodemographic Characteristics of FCs

A self-designed questionnaire was used to collect sociodemographic characteristics of FCs including gender, age, education, marital status, relationship with patients, and length of care.

#### Impact of Events Scale-Revised

PTSS was assessed using the Chinese version IES-R, which was developed by Weiss and Marmar (18) and modified by SuRan (19). It consists of 22 items and includes three dimensions: avoidance, intrusion, and hyperarousal. Each item has a score range of 0–4, with higher scores reflecting higher levels of PTSS. The Cronbach's α was 0.89 and the split-half reliability was 0.93(19). Cronbach's α in the current sample was 0.77.

#### Uncertainty in Illness Scale Family Caregiver Version

Caregivers' IU was assessed using the Chinese version UIS-FC, which was developed by Mishel (20) and modified by Hongyan (21). It consists of 30 items and includes four dimensions: unpredictability, ambiguity, complexity, and lack of informativeness. These items were scored on five-point Likert scales. The total score ranges from 30 to 150, with higher scores indicating higher levels of IU. The content validity index (CVI) was 0.87 and Cronbach's α was 0.89 (21). Cronbach's α in the current sample was 0.81.

#### Anticipatory Grief Scale

The AG was assessed using the Chinese version AGS, which was developed by Theut (22) and modified by Dajun (23). It consists of 27 items and includes seven dimensions: sadness, feelings of loss, anger, irritability, guilt, anxiety, and ability to complete tasks. These items were scored on five-point Likert scales. Higher scores reflect higher levels of AG. The CVI was 0.96 and Cronbach's α was 0.90 (23). Cronbach's α in the current sample was 0.91.

### Statistical Analysis

The IBM SPSS Statistics 26.0 (IBM Corp., USA) was used for data analysis. The sociodemographic characteristics of the participants were examined by computing frequencies and percentages. One-way ANOVAs and t tests were used to determine the relationship between FCs' characteristics and the three variables, and Pearson correlations were used to test for unadjusted associations between variables. Statistical significance was set at 0.05. The hypothesized model was tested using SEM with IBM SPSS AMOS version 26.0 (IBM Corp., USA). The maximum-likelihood estimation of the entire system in a hypothesized model, and enables the assessment of variables with the data (24). In our analysis, the measurement model was confirmed using confirmatory factor analysis (CFA), and then we performed SEM analysis to measure the fit and path coefficients of the structural model. The model fit indices were as follows: Chi-square (χ^2^), degrees of freedom (df), value of χ^2^/df, goodness-of-fit index (GFI), adjusted GFI (AGFI), comparative fit index (CFI), Tucker-Lewis index (TLI), root mean square error of approximation (RMSEA), and standardized root mean square residual (SRMR). The recommended value for GFI, AGFI, CFI, and TLI is 0.90 or higher. The RMSEA would be “close to” 0.09 or lower, SRMR would be “close to” 0.05 or lower, and χ^2^/df would be “close to” 5.00 or lower, indicating a good model fit (25). Finally, we used the bootstrap test to measure the direct, indirect and total effects of the structural model (26).

### Ethical Considerations

The study was approved by the Ethics Committee of Liaoning Cancer Hospital and Institute. Based on the Declaration of Helsinki, participants had the right to leave the study at any time. Written informed consent was obtained from all participants.

## Results

### Sociodemographic Characteristics

A total of 254 FCs of patients with advanced lung cancer participated in this study. The majority were females (63.4%), age 36–59 years (58.7%), high school (53.1%), married (85.0%), parents of the patients (52.4%), and length of care <6 months (58.7%).

### PTSS, IU and AG, According to Sample Characteristics

Female FCs reported significantly higher IES-R, UIS-FC, and AGS scores than male (*P* < 0.05 or 0.001). FCs aged ≤35 years reported significantly higher IES-R and UIS-FC scores than those aged 36–60 years and ≥60 years (*P* < 0.05). FCs with a bachelor's degree and above reported higher IES-R scores than those with high school education, primary school education and below (*P* < 0.05). FCs who were married reported lower IES-R, UIS-FC, and AGS scores than other (*P* < 0.05 or <0.01). Regarding relationships with patients, spouses reported lower IES-R, UIS-FC, and AGS scores than compared with parents and children (*P* < 0.05, 0.01 or 0.001). There were significant differences in the AGS scores for FCs with different lengths of care (*P* < 0.05) (Supplementary Table 1).

### The Interrelationships Between PTSS, UI and AG

AG were significantly and positively correlated with PTSS. The Pearson's correlation coefficients ranged from 0.25 to 0.62 (*P* < 0.01). Additionally, AG were significantly correlated with UI. The Pearson's correlation coefficients ranged from 0.13 to 0.54 (*P* < 0.01 or 0.05) (Table 1). The PTSS were significantly and positively associated with UI. The correlation coefficients ranged from 0.20 to 0.52 (*P* < 0.01).

**Table 1:** The interrelationships between PTSS, IU and AG (r).

**AGS scores**	**IES-R scores**				**UIS-FC scores**				
	**Total**	**AVO**	**INT**	**HYP**	**Total**	**UNP**	**AMB**	**COM**	**LAI**
Total	0.56^**^	0.62^**^	0.38^**^	0.38^**^	0.54^**^	0.52^**^	0.45^**^	0.31^**^	0.50^**^
SAD	0.49^**^	0.52^**^	0.33^**^	0.35^**^	0.47^**^	0.44^**^	0.41^**^	0.26^**^	0.41^**^
FOL	0.46^**^	0.49^**^	0.33^**^	0.31^**^	0.45^**^	0.43^**^	0.38^**^	0.28^**^	0.36^**^
ANG	0.46^**^	0.50^**^	0.34^**^	0.28^**^	0.45^**^	0.42^**^	0.37^**^	0.28^**^	0.43^**^
IRR	0.44^**^	0.50^**^	0.26^**^	0.32^**^	0.45^**^	0.45^**^	0.38^**^	0.27^**^	0.43^**^
GUI	0.45^**^	0.51^**^	0.31^**^	0.30^**^	0.47^**^	0.45^**^	0.41^**^	0.25^**^	0.41^**^
ANX	0.45^**^	0.51^**^	0.28^**^	0.33^**^	0.43^**^	0.42^**^	0.34^**^	0.23^**^	0.42^**^
ACT	0.40^**^	0.38^**^	0.26^**^	0.25^**^	0.31^**^	0.31^**^	0.26^**^	0.13^*^	0.35^**^

^*^P < 0.05, ^**^P < 0.01. PTSS, Post-traumatic Stress Symptoms; IU, Illness Uncertainty; AG, Anticipatory Grief; IES-R, Impact of Events Scale-Revised; UIS-FC, Uncertainty in Illness Scale Family Caregiver Version; AGS, Anticipatory Grief Scale; AVO, Avoidance; INT, Intrusion; HYP, Hyperarousal; UNP, Unpredictability; AMB, Ambiguity; COM, Complexity; LAI, Lack of Informativeness; SAD, Sadness; FOL, Feelings of Loss; ANG, Anger; IRR, Irritability; GUI, Guilt; ANX, Anxiety; ACT, Ability To Complete Tasks.

### Reliability and Validity of the Measurement Model

To measure the internal consistency reliability, convergent validity and discriminant validity of the constructs in our hypothetical model, we performed CFA on the three constructs of PTSS, IU, and AG (Table 2). The results indicated that the composite reliability (C.R.) of each construct ranged from 0.765 to 0.910, exceeding the C.R. threshold value of 0.60 (27), and providing evidence of internal consistency reliability. In addition, the standardized factor loadings of the individual dimensions in the model were between 0.672 and 0.805, exceeding threshold value of 0.50 (27), and reached significant (all *P* < 0.001), giving preliminary evidence for the convergent validity of the measurement model. Meanwhile, the average variance extracted (AVE) of all constructs ranged from 0.517 to 0.592, exceeding the AVE threshold value of 0.50 (27), and thus the convergent validity was acceptable. Moreover, the estimated intercorrelations among all constructs were less than the square roots of the AVE in each construct, and this provided support for discriminant validity (28) (Table 3).

**Table 2:** CFA for the measurement model.

**Construct**	**Variable**	**Unstandardized**	**S.E**.	**t-value**	**P**	**Standardized**	**SMC**	**C.R**.	**AVE**
		**factor loadings**				**factor loadings**			
PTSS	AVO	1.000				0.672	0.452	0.765	0.522
	INT	1.134	0.135	8.368	^***^	0.747	0.558		
	HYP	0.982	0.117	8.370	^***^	0.745	0.555		
IU	UNP	1.000				0.712	0.507	0.811	0.517
	AMB	2.991	0.311	9.607	^***^	0.723	0.523		
	COM	1.241	0.134	9.258	^***^	0.688	0.473		
	LAI	1.181	0.120	9.845	^***^	0.752	0.566		
AG	SAD	1.000				0.730	0.533	0.910	0.592
	FOL	1.311	0.115	11.425	^***^	0.736	0.542		
	ANG	0.956	0.076	12.495	^***^	0.803	0.645		
	IRR	0.937	0.075	12.526	^***^	0.805	0.648		
	GUI	0.899	0.077	11.705	^***^	0.754	0.569		
	ANX	1.039	0.085	12.276	^***^	0.789	0.623		
	ACT	0.785	0.066	11.849	^***^	0.763	0.582		

*** *P < 0.001. CFA, Confirmatory factor analysis; S.E, Standard errors; SMC, Squared Multiple Correlations; C.R., Composite reliability;AVE, Average variance extracted; PTSS, Post-traumatic Stress Symptoms; IU, Illness Uncertainty; AG, Anticipatory Grief; AVO, Avoidance; INT, Intrusion; HYP, Hyperarousal; UNP, Unpredictability; AMB, Ambiguity; COM, Complexity; LAI, Lack of Informativeness; SAD, Sadness; FOL, Feelings of Loss; ANG, Anger; IRR, Irritability; GUI, Guilt; ANX, Anxiety; ACT, Ability To Complete Tasks*.

**Table 3:** Discriminant validity analysis for the measurement model.

	**AVE**	**PTSS**	**IU**	**AG**
PTSS	0.522	**0.722**		
IU	0.517	0.684	**0.719**	
AG	0.592	0.720	0.661	**0.769**

*AVE, Average variance extracted; PTSS, Post-traumatic Stress Symptoms; IU, Illness Uncertainty; AG, Anticipatory Grief. The bold values indicate the square root of AVE for discriminant validity*.

As with all self-reported data there is a potential for common method variance (CMV) resulting from multiple sources (29). Therefore, we performed statistical analyses to assess the severity of CMV. First, a Harmon one-factor test was conducted on the 14 crucial variables in our hypothetical model (30). Following Jukka, the first factor tends to explain over half of the variance indicating the presence of CMV (31). Results illustrated that 14 factors are present and the most covariance explained by one factor is 43.67 percent, showing that CMV are not a likely contaminant of our results. Second, we included in the model 1 a common method factor whose indicators included all the principal constructs' indicators. Subsequently, we interconnected the CFA constructs into fully correlated constructs in model 2. If method variance is largely responsible for the covariation among the measures, df and χ^2^ values of difference between model 1 and model 2 should indicate that there was no significant (30, 32). Given the difference between two models did demonstrate the significance, we contend that the CMV is unlikely to be a serious concern for this study. (Δχ^2^= 221.065, Δdf = 3, *P* < 0.001).

### Test of the Structural Model

The structural modeling results showed that the hypothesized model fit the data well (χ^2^ = 124.507, df = 74, χ^2^/df = 1.683, GFI = 0.937, AGFI = 0.910, CFI = 0.971, TLI = 0.964, RMSEA = 0.052, SRMR = 0.047). We use a causal step strategy to investigate the first mediation condition with respect to hypothesis 1 (33). As shown in Table 1, the correlation coefficients indicated that PTSS was significantly and positively associated with AG (r = 0.56, *P* < 0.01). In addition, the results of the direct effect of PTSS on AG (standardized direct effect = 0.76, *P* < 0.001) was statistically significant. Therefore, hypothesis 1 was supported.

**Figure 1:**
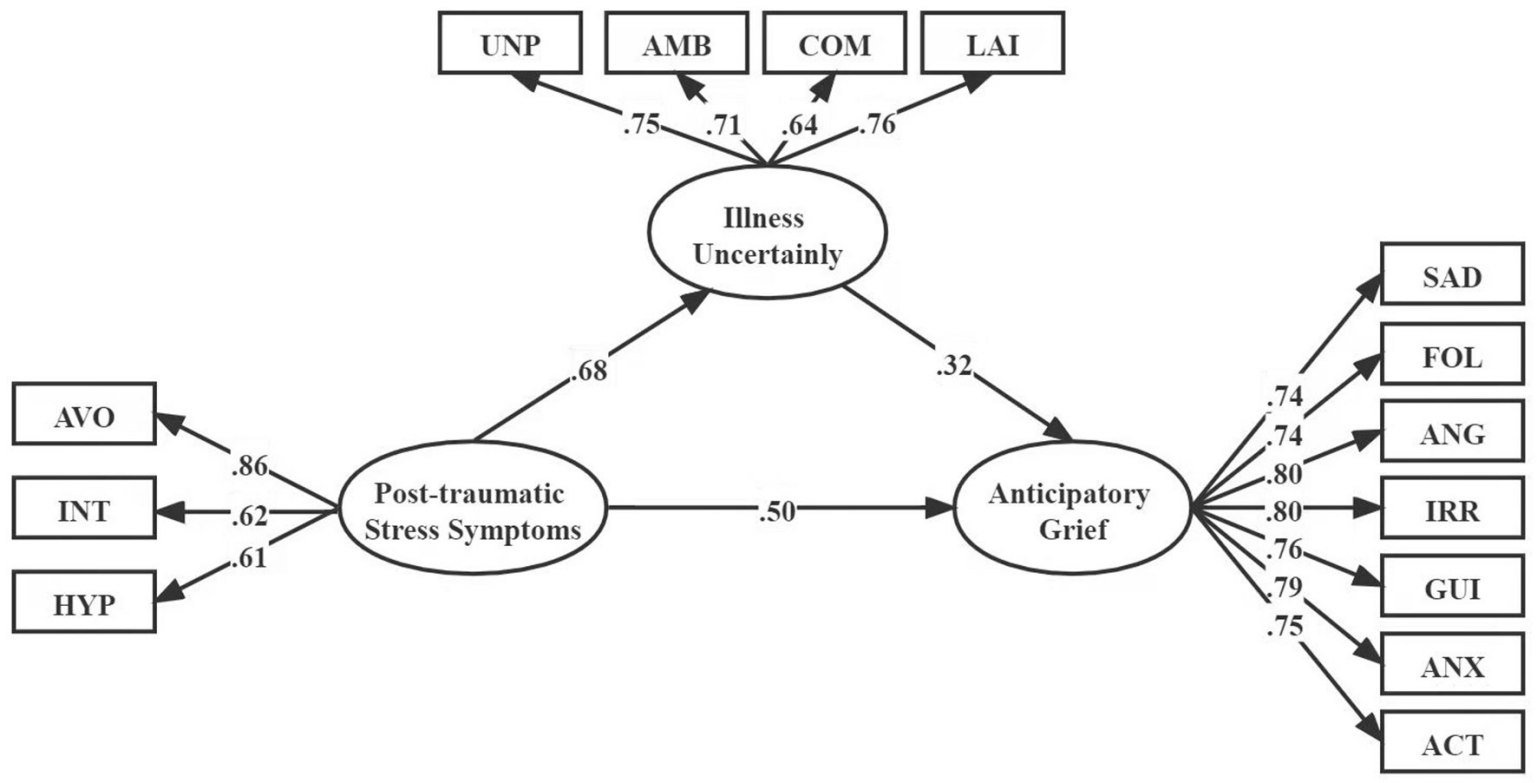
Structural equation modeling of the hypothesized model. AVO, Avoidance; NT, Intrusion; HYP, Hyperarousal; UNP, Unpredictability; AMB, Ambiguity; COM, Complexity; LAI, Lack of Informativeness; SAD, Sadness; FOL, Feelings of Loss; ANG, Anger; IRR, Irritability; GUI, Guilt; ANX, Anxiety; ACT, Ability To Complete Tasks.

To test hypothesis 2, we measured the second condition of mediation. The correlation coefficients indicated that PTSS was significantly and positively associated with IU (r = 0.48, *P* < 0.01), IU was significantly and positively associated with AG (r = 0.54, *P* < 0.01). In addition, the results of the direct effects of PTSS on IU (standardized direct effect = 0.50, *P* < 0.001), and the direct effect of IU on AG (standardized direct effect = 0.32, *P* < 0.001), were all statistically significant (Figure 1). To examine the indirect effects of the dependent variable through the mediator, we performed bias-corrected percentile bootstrapping and percentile bootstrapping at a 95% confidence interval with 1,000 bootstrap samples (Table 4) (34). We calculated the confidence interval of the lower and upper bounds to examine of whether the indirect effects were significant (28). The result of the bootstrap test confirmed the existence of a significant and positive mediating effect for IU between PTSS and AG (indirect effect = 0.17, *P* < 0.01). Hypotheses 2 was thus supported.

**Table 4:** Direct, indirect, and total effects of the hypothesized model.

	**Point estimate**	**Product of coefficients**		**Bootstrapping**				
				**Bias-Corrected 95% CI**		**Percentile 95% CI**		**Two-tailed significance**
		**SE**	**Z**	**Lower**	**Upper**	**Lower**	**Upper**	
Direct effects								
PTSS → AG	0.391	0.081	4.827	0.256	0.570	0.249	0.567	0.002^**^
Indirect effects								
PTSS → AG	0.168	0.051	3.294	0.071	0.273	0.064	0.262	0.005^**^
Total effects								
PTSS → AG	0.560	0.063	8.889	0.437	0.686	0.439	0.688	0.002^**^

Estimating of 1,000 bootstrap sample, ^**^P < 0.01. PTSS, Post-traumatic Stress Symptoms; AG, Anticipatory Grief.

## Discussion

FCs assume the primary responsibility for the care of patients with advanced cancer in China. However, due to the irreversible nature of advanced cancer patients' disease, FCs may experience varying degrees of AG when faced with the imminent death of the patient (15). AG not only affects the FCs 's ability to assess the patient's care needs, but also leads to a decrease in the quality of palliative care. In addition, experiencing AG may also have a negative impact on the FCs 's mental state, predisposing them to negative emotions such as anger, fear, and guilt and self-blame (6). Nevertheless, there is still limited evidence of study on FCs with advanced cancer experiencing AG. Therefore, in view of the potentially serious adverse outcomes for FCs with AG, a more in-depth exploration of the mechanisms underlying the development of AG is necessary to inform the precise implementation of clinical interventions.

The present study found significant differences in IES-R, UIS-FC and AGS when PTSS, IU and AG were examined according to sociodemographic characteristics. Culturally, women are the primary source of caregiving. Meanwhile, female FCs have been found to be more likely to use emotion-focused coping strategies. Therefore, it was not amazing to find that women reported significantly greater PTSS, IU and AG than men. In this study, FCs aged <35 years reported higher levels of PTSS and IU. Similarly, Elisavet et al. found that younger age was significantly associated with higher PTSS (35). Heleen et al. reported that the age of mother caregiver of children with cancer was negatively associated with uncertainty (36). Young FCs may face the stress of work and economic and social challenges along with their caregiving responsibilities. When work-life balance is difficult it can lead to physical and psychological symptoms, which may be one reason why younger FCs may have higher levels of PTSS and IU, while older homemakers have more positive emotional responses. In terms of education, FCs with at least a bachelor's degree reported higher levels of PTSS. One possible explanation is that FCs with higher education possess more but not deeper information and knowledge about cancer treatment and nursing and can be highly alert to any subtle symptoms or even normal reactions of the patients, which may increase their PTSS. However, there was no significant difference in education level in terms of IU and AG. In addition, our results show that married FCs reported lower levels of PTSS, IU, and AG because they are more likely to have partner support. In terms of the relationship with the patient, the patient's spouse had lower levels of PTSS, IU and AG than the patient's children and parents. This is inconsistent with the results of previous study and the reason may be the influence of blood relationship (37). In China, there is a traditional belief that couples are not blood relationship, but are legal relationship. In fact, many couples are not selfless; they look more to their own interests and do not think to take on problems together when they come up. Furthermore, FCs with <6 months of care had higher AG levels. Burton et al. study showed that caregivers who spent fewer months caring for the patient before the bereavement would increase the level of grief (38). FCs in the short term do not cope well with the fact of patient's illness and are not adapted to the life of intense care. AG level can therefore exacerbate.

We found a possible causal relationship between PTSS, IU and AG and FCs in 254 patients with advanced lung cancer based on the theoretical basis and SEM analysis. The results demonstrate PTSS had a direct effect on AG. Meanwhile, IU was shown to have a mediation effect in FCs' PTSS and AG. In a full mediation model, the direct effect of PTSS would have become insignificant when the role of IU was added to the model. The model illustrates only partial mediation as the path between PTSS and AG remains significant in the full model. Overall, the results explain well the hypotheses proposed in this study.

The first aim of this study was to examine the associations between PTSS and AG in FCs of patients with advanced lung cancer. Consistent with previous studies (11), the present study showed that these associations are positive and statistically significant. Advanced lung cancer is a traumatic event for FCs, who can develop a variety of post-traumatic symptoms due to the changes in the cancer and the patient's uncomfortable response. As patients require frequent hospitalization, FCs are constantly in trauma-related scenarios while caring for the patient, and they unconsciously repeat all the information about the cancer and exhibit intrusive thinking. Some FCs will avoid cancer-related topics, not wanting to face the deterioration of the patient's cancer and displaying fear for the patient after death. FCs become hyperarousal to any subtle, even normal changes and reactions in the patient's body. These post-traumatic symptoms can lead FCs to perceive that they may lose a loved one to cancer and develop AG, or even psychological illness and suicidal tendencies. Therefore, to reduce the level of AG in FCs, this can be achieved by reducing the level of PTSS and reducing the intrusion, avoidance and hyperarousal of stressful events. It is suggested that healthcare professionals can use a variety of psychotherapeutic interventions to minimize the adverse effects of intrusive thinking and to help FCs to properly deal with the deterioration of the patient's illness and death.

The second aim was to determine to what extent IU mediates the association between PTSS and AG. The significance of the finding is that intervening in IU is an important strategy to alleviate AG. The present study showed that IU scores of FCs of patients with advanced lung cancer was positively correlated with PTSS and AG scores. FCs often have the stressful event of the patient's cancer in their minds and are reluctant to face reality and avoid any cancer-related information, which may lead to a lack of information for FCs and further interfere with their ability to acquire health-related knowledge. Due to the lack of information and ability, FCs do not predict the course of cancer, coupled with the various complex treatments, which these uncertainty factors often overwhelm FCs and thus deepen IU. IU can further increase the physiological and psychological stress of FCs, reducing their confidence in coping with and overcoming traumatic events. Moreover, FCs are at risk of losing a loved one, which makes the experience of caring for a palliative care patient unique and complex, ultimately leading to AG for FCs. Therefore, AG levels can be mitigated by reducing the IU of FCs. It is recommended that healthcare professionals should provide more care and communication to FCs, disseminate cancer-related knowledge, and satisfy FCs' information needs. It is also necessary to strengthen FCs' mental health education, assist them in establishing a correct concept of death, understand that death is an inevitable law of nature, and help them cope with the pain and grief of the impending loss of a close relative.

There are still some limitations in this study. First, the participants in this study were all from the same hospital and a convenience sampling method was used, thus limiting generalization due to potential selection bias. In addition, this study is a preliminary cross-sectional study does not enable causal inferences to be made yet. Although we used SEM to conduct a simultaneous testing of our proposed model in FCs of advanced lung cancer patients, the results still need to be treated with caution. It is desirable that future longitudinal studies will be conducted to further clarify the causal relationship between them.

## Conclusion

FCs need practical and emotional support to deal with the mental trauma they endure while providing palliative care. FCs of advanced lung cancer patients may experience reduced quality of care due to AG, and interventions for FCs from the perspective of their PTSS and IU are recommended.

## Data Availability Statement

The raw data supporting the conclusions of this article will be made available by the authors, without undue reservation.

## Ethics Statement

The studies involving human participants were reviewed and approved by Liaoning Cancer Hospital and Institute. The patients/participants provided their written informed consent to participate in this study.

## Author Contributions

DS, ZM, XZ, and LZ: material preparation, data collection, and analysis were performed. DS and ZM: the first draft of the manuscript was written. JL commented on previous versions of the manuscript. All authors contributed to the study conception, design, read, and approved the final manuscript.

## Funding

This work was supported by the Research Project on Humanities and Social Sciences of Liaoning University of Traditional Chinese Medicine (2021LNZYQN007).

## Conflict of Interest

The authors declare that the research was conducted in the absence of any commercial or financial relationships that could be construed as a potential conflict of interest.

## Publisher's Note

All claims expressed in this article are solely those of the authors and do not necessarily represent those of their affiliated organizations, or those of the publisher, the editors and the reviewers. Any product that may be evaluated in this article, or claim that may be made by its manufacturer, is not guaranteed or endorsed by the publisher.
