# Supplementary material for: Relationship Between Post-traumatic Stress Symptoms and Anticipatory Grief in Family Caregivers of Patients With Advanced Lung Cancer: The Mediation Role of Illness Uncertainty
Source: Front Psychiatry. 2022 Jun 9;13:914862. doi: 10.3389/fpsyt.2022.914862 (PMC9218190; doi:10.3389/fpsyt.2022.914862)
Supplement: Supplementary file 1 [file Table_1.DOCX]

Table S1. PTSS, IU and AG, according to sociodemographic characteristics of FCs

| Caregiver Outcomes | IES-R | UIS-FC | AGS |
| --- | --- | --- | --- |
| Gender |  |  |  |
| Male | 41.72±6.80 | 82.80±12.59 | 77.10±15.22 |
| Female | 44.24±8.38 | 86.22±12.96 | 84.92±15.47 |
| *t* | 2.464 | 2.048 | 3.905 |
| *P* | .014* | .042* | .000*** |
| Age |  |  |  |
| ≤35 | 45.37±9.24^a^ | 88.04±14.20^a^ | 84.92±17.61 |
| 36-59 | 42.64±6.82 | 84.15±12.10 | 80.95±14.87 |
| ≥60 | 42.00±8.32 | 82.12±12.70 | 80.91±15.54 |
| *F* | 3.463 | 3.197 | 1.622 |
| *P* | .033* | .043* | .200 |
| Education |  |  |  |
| Primary school or less | 39.36±6.02 | 85.00±9.56 | 79.55±17.21 |
| High school | 42.44±8.25 | 83.77±13.57 | 80.96±15.47 |
| Bachelor and above | 44.81±7.41^a^ | 86.45±12.28 | 83.69±16.08 |
| *F* | 4.204 | 1.299 | 1.040 |
| *P* | .016* | .275 | .355 |
| Marital status |  |  |  |
| Married | 42.81±7.67 | 84.00±12.38 | 80.96±15.84 |
| Other | 46.21±8.73 | 90.47±14.58 | 88.26±14.29 |
| *t* | 2.469 | 2.894 | 2.657 |
| *P* | .014* | .004** | .008** |
| Relationship with patients |  |  |  |
| Spouse | 41.09±7.74^b^ | 82.26±11.81^b^ | 76.73±16.29^b^ |
| Parents | 44.77±7.25 | 86.57±13.38 | 84.64±14.36 |
| Children | 43.69±9.88 | 86.17±13.09 | 87.10±16.46 |
| *F* | 6.178 | 3.231 | 9.019 |
| *P* | .002** | .041* | .000*** |
| Length of care |  |  |  |
| ＜6 months | 43.89±7.66 | 85.58±12.39 | 83.67±14.76^a^ |
| 6-12 Months | 41.27±9.67 | 81.08±15.64 | 76.00±17.03 |
| ＞12 Months | 43.16±7.32 | 85.74±12.22 | 81.81±16.74 |
| *F* | 1.652 | 1.978 | 3.573 |
| *P* | .194 | .140 | .030* |

Note: **P* < .05, ***P* < .01, ****P*< .001. ^a^Post-hoc tests showed the score of this group was higher than the scores of the other groups. ^b^Post-hoc tests showed the score of this group was lower than the scores of the other groups. PTSS = Post-traumatic Stress Symptoms, IU= Illness Uncertainty, AG= Anticipatory Grief, FCs= Family Caregivers, IES-R = Impact of Events Scale-Revised, UIS-FC = Uncertainty in Illness Scale Family Caregiver Version, AGS = Anticipatory Grief Scale.
