# Supplementary figures and images for: Relationship Between Post-traumatic Stress Symptoms and Anticipatory Grief in Family Caregivers of Patients With Advanced Lung Cancer: The Mediation Role of Illness Uncertainty
Source: Front Psychiatry. 2022 Jun 9;13:914862. doi: 10.3389/fpsyt.2022.914862 (PMC9218190; doi:10.3389/fpsyt.2022.914862)

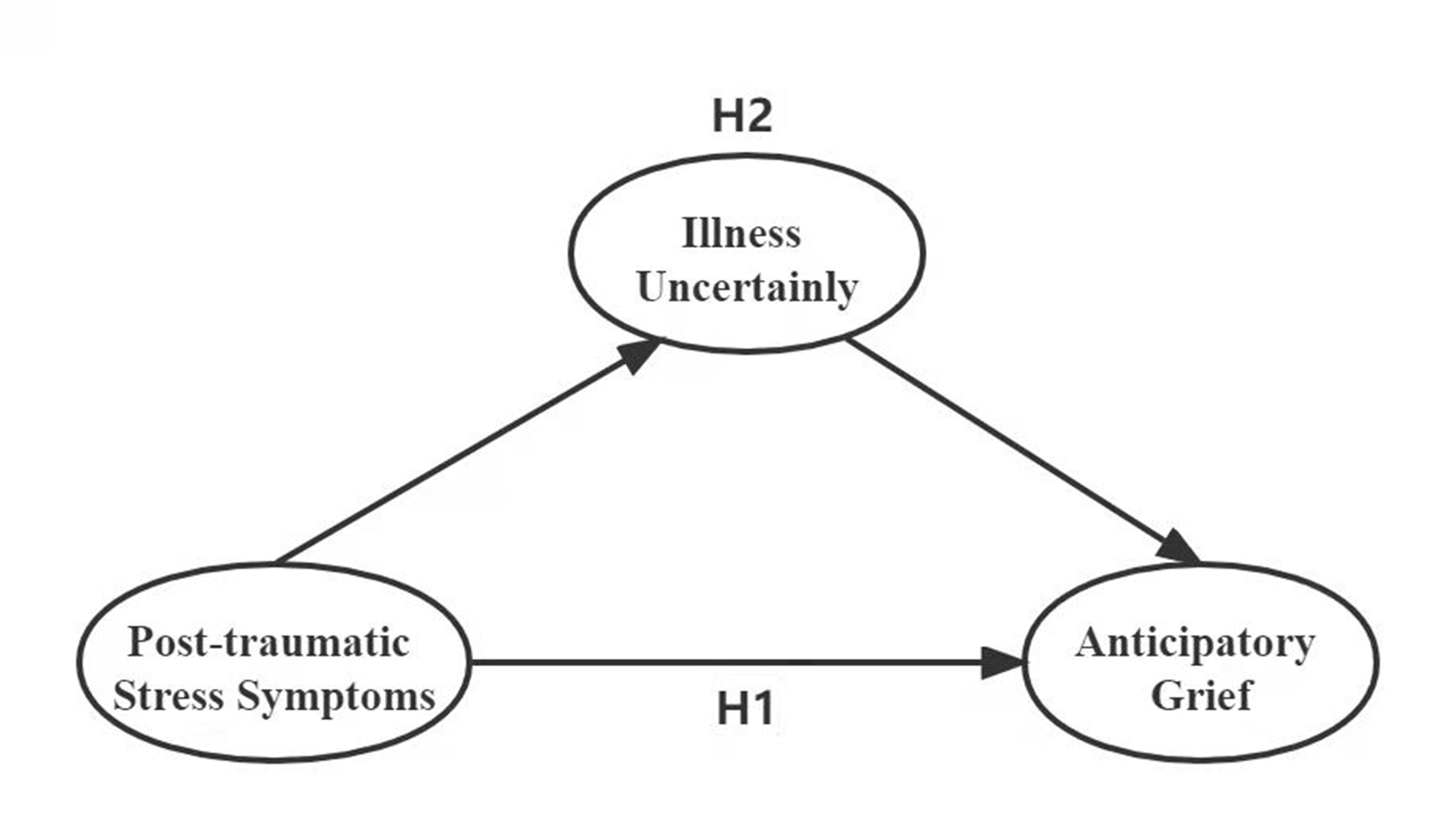

Supplement: Supplementary file 2 [file Image_1.TIF]
